# Effects of Consuming Pulsed UV Light-Treated *Pleurotus citrinopileatus* on Vitamin D Nutritional Status in Healthy Adults

**DOI:** 10.3390/foods14020259

**Published:** 2025-01-15

**Authors:** Chih-Ching Hsu, Chiao-Ming Chen, Yu-Ming Ju, Yu-Ching Wu, Huei-Mei Hsieh, Shu-Hui Yang, Chien-Tien Su, Te-Chao Fang, Widiastuti Setyaningsih, Sing-Chung Li

**Affiliations:** 1 School of Nutrition and Health Sciences, College of Nutrition, Taipei Medical University, Taipei 11031, Taiwan; ga56110007@tmu.edu.tw; 2 Department of Food Science, Nutrition, and Nutraceutical Biotechnology, Shih Chien University, Taipei 10462, Taiwan; charming@g2.usc.edu.tw; 3 Institute of Plant and Microbial Biology, Academia Sinica, Taipei 11529, Taiwan; yumingju@gate.sinica.edu.tw (Y.-M.J.); saseki@gate.sinica.edu.tw (Y.-C.W.); monicah@gate.sinica.edu.tw (H.-M.H.); 4 Taiwan Agricultural Research Institute, Fengshan Tropical Horticultural Experiment Branch, Kaohsiung City 83052, Taiwan; debbie@tari.gov.tw; 5 Department of Family Medicine, Taipei Medical University Hospital, Taipei 11031, Taiwan; ctsu@tmu.edu.tw; 6 School of Public Health, College of Public Health, Taipei Medical University, Taipei 11031, Taiwan; 7 Division of Nephrology, Department of Internal Medicine, School of Medicine, College of Medicine, Taipei Medical University, Taipei 11031, Taiwan; fangtc@tmu.edu.tw; 8 Division of Nephrology, Department of Internal Medicine, Taipei Medical University Hospital, Taipei Medical University, Taipei 11031, Taiwan; 9 Taipei Medical University-Research Center of Urology and Kidney, Taipei Medical University, Taipei 11031, Taiwan; 10 Department of Food and Agricultural Product Technology, Faculty of Agricultural Technology, Universitas Gadjah Mada, Jalan Flora, Depok, Sleman, Yogyakarta 55281, Indonesia; widiastuti.setyaningsih@ugm.ac.id

**Keywords:** vitamin D_2_, ergosterol, *Pleurotus citrinopileatus*, pulsed ultraviolet light, 25(OH)D_2_, intact parathyroid hormone

## Abstract

Vitamin D, essential for growth and health, is often deficient in Taiwan despite abundant sunlight. Plant-derived vitamin D_2_ (ergocalciferol) is bioavailable, environmentally friendly, and cost-effective. This study evaluated the efficacy of enhancing *Pleurotus citrinopileatus* (PC) mushrooms’ vitamin D_2_ content through pulsed ultraviolet (PUV) light and its impact on vitamin D status in humans. In a four-week randomized parallel trial, 36 healthy participants were assigned to three groups: a control group, a group consuming 10 g/day PUV-treated PC (PC-10 g), and a group consuming 100 g/day PUV-treated PC (PC-100 g). Blood samples collected pre- and post-intervention measured serum 25(OH)D_2_, 25(OH)D_3_, and biochemical parameters. After four weeks, serum 25(OH)D_2_ levels significantly increased in the PC-10 g group (1.47 ± 1.42 ng/mL to 9.50 ± 7.10 ng/mL, *p* = 0.001) and in the PC-100 g group (1.94 ± 2.15 ng/mL to 21.82 ± 16.75 ng/mL, *p* = 0.002), showing a 10.2-fold rise. The PC-100 g group also experienced a 37.6% reduction in serum intact parathyroid hormone (I-PTH) levels (26.26 ± 9.84 pg/mL to 16.38 ± 5.53 pg/mL). No adverse effects were reported. PUV-treated PC mushrooms significantly increase serum 25(OH)D_2_ levels and reduce I-PTH, particularly at higher doses. These findings underscore the potential of vitamin-D-enriched PC as a sustainable, fungi-derived food source for addressing vitamin D deficiency.

## 1. Introduction

Vitamin D, a steroid hormone, is a vital nutrient for humans, playing crucial roles in bone growth, calcium and phosphorus balance, and immune regulation. Vitamin D is classified into two types based on its source: vitamin D_2_ (ergocalciferol) and vitamin D_3_ (cholecalciferol). Vitamin D_2_ primarily comes from fungi-derived sources, while vitamin D_3_ is derived from animal-based sources or synthesized in the skin through sunlight exposure. Ergosterol, a fungi-derived precursor of vitamin D_2_, can be converted into ergocalciferol through ultraviolet light activation and subsequently metabolized into its active form for utilization in the body [1,2,3].

Although Taiwan is in the subtropical zone and generally experiences ample sunlight, a high proportion of the population still suffers from vitamin D deficiency. Lee et al. reported that among participants aged ≥ 30 years without chronic kidney disease (CKD) in northern Taiwan, 22.4% of the study population had vitamin D deficiency (25(OH)D level < 20 ng/mL). Additionally, the study observed a significantly higher prevalence of vitamin D deficiency in women compared to men [4]. Due to the health and environmental benefits associated with vegetarianism, the vegetarian population in Taiwan has been increasing. Several studies have shown that vegetarians and vegans exhibit the highest prevalence of vitamin D deficiency [5,6]. Similarly, a small study in Taiwan reported that individuals adhering to a strict vegetarian diet had significantly lower vitamin D levels compared to non-vegetarians [7].

Mushrooms naturally contain high concentrations of ergosterol in their cell walls. Upon exposure to ultraviolet (UV) irradiation, ergosterol is converted into previtamin D_2_, which subsequently undergoes a temperature-dependent thermal isomerization process to form ergocalciferol, also known as vitamin D_2_ [8]. However, most fresh retail mushrooms are cultivated in atmospherically controlled growing rooms under dark conditions, resulting in a vitamin D_2_ content typically less than 1 μg per 100 g of fresh weight (FW) [9]. To produce nutritionally relevant levels of vitamin D_2_, mushrooms can be exposed to controlled UV irradiation using fluorescent or pulsed UV lamps. This exposure can be applied during their growth stage, after harvest, or following the drying process [10].

Since vitamin D_2_ is primarily found in fungi and yeast, common fruits and vegetables, which lack ergosterol, cannot serve as a source of vitamin D_2_ intake [11]. *Pleurotus citrinopileatus* (PC) is a nutritionally valuable edible fungus rich in bioactive compounds, including proteins, polysaccharides, amino acids, minerals, dietary fiber, and trace elements. It is widely consumed in Taiwan [12] and cultivated globally, with East Asia and northeastern China as major production regions [13]. Huang et al. investigated vitamin D_2_ production in various mushroom species exposed to UVB irradiation and found that PC exhibited the highest conversion efficiency. In PC, the vitamin D_2_ content increased significantly from 3.93 ± 0.44 µg/g to 208.65 ± 6.08 µg/g dry matter after 2 h of UVB exposure, representing an approximate 53-fold increase [14].

Several studies have explored the effects of UVB irradiation on mushroom quality. While UVB exposure enhances nutritional components such as phenolics, flavonoids, and vitamin D_2_, it also leads to quality deterioration, including darkening, texture changes, and moisture exudation. These issues adversely affect the appearance, marketability, and shelf-life of mushrooms [15,16,17]. In contrast, Kalaras and Koyyalamudi studied the effects of postharvest pulsed ultraviolet (PUV) light on white button mushrooms (*Agaricus bisporus*), finding that PUV treatment significantly boosted vitamin D_2_ levels without compromising appearance, texture, or shelf-life, making it an effective method for enhancing nutritional value while preserving quality [10,18].

This study explores the effectiveness of PUV light in enhancing the vitamin D_2_ content in PC mushrooms and its impact on healthy adult vitamin D status. It evaluates whether PUV can increase vitamin D_2_ levels and improve nutritional status after four weeks of intake and whether the rise in blood vitamin D levels is attributed to increased 25(OH)D_2_ concentrations, highlighting the novelty of this research.

## 2. Materials and Methods

### 2.1. PUV Light Treatment PC

The fresh PC mushrooms used in this experiment were cultivated by a professional mushroom farmer in Yuchi Township, Nantou County. The PUV light treatment was performed by Dr. Yang from the Kaohsiung-Fengshan Tropical Horticulture Experiment Office. Freshly harvested PC mushrooms were exposed to PUV light using a Xenon Z-1000-OEM device (MOS Technology Inc., Hsinchu, Taiwan). The equipment had an energy specification of 1516 J/s with a flash frequency of 3 Hz. The irradiated area was 30 cm × 8 cm = 240 cm^2^, resulting in an energy density of 6.31 J/s·cm^2^. Each square centimeter of the mushrooms was exposed for 1.5 s, delivering a total energy of 9.465 J/cm^2^. This treatment was optimized to enhance the vitamin D_2_ content of the mushrooms while preserving their quality.

### 2.2. Preparation of PC Instant Meal Packs for Participants

PC mushrooms treated with PUV light were transported under refrigeration to the experimental kitchen at Shih Chien University. After cleaning, the mushrooms were steamed for 20 min, seasoned with vegetable oil and spices, vacuum-sealed, and frozen at −20 °C. The preparation process was carried out weekly to ensure quality, with PC instant meal packs prepared in two portions: 10 g and 100 g. Frozen mushroom meal packs were distributed to participants in weekly portions, with instructions to heat them using a water bath before consumption. Participants were instructed to consume one pack daily with a meal.

### 2.3. Extraction and Analysis of Vitamin D_2_ in PC Mushrooms

Vitamin D_2_ was extracted and analyzed following the method of Huang et al. (2015) with modifications [14]. The vitamin D_2_ content in the PC exposed to PUV light was measured at two stages: before cooking and after cooking. The process began by freeze-drying the PC, followed by weighing 5 g of the powdered sample into a centrifuge tube. Next, 10 mL of dimethyl sulfoxide (DMSO) was added, and the mixture was sonicated in a water bath at 40–50 °C for 30 min. Subsequently, 10 mL of a methanol solution (1:1, *v*/*v*) was added and thoroughly mixed. Afterward, 20 mL of n-hexane was introduced, and the mixture was agitated for 30 min. The sample was centrifuged at 3500 rpm for 10 min, and the upper layer was collected into a concentration flask. The lower layer was re-extracted twice more with 20 mL of n-hexane. The combined upper layers were concentrated to near dryness under reduced pressure in a water bath at 30–40 °C. The residue was dissolved in n-hexane and diluted to 5 mL. The final solution was filtered through a membrane filter for analysis.

Vitamin D_2_ analysis in the PC mushrooms was performed using a Waters^®^ ACQUITY UPLC system (Water, Milford, CT, USA) equipped with a Vydac C18 column (5 μm, 4.6 × 250 mm). The detection wavelength was set at 265 nm, with a mobile phase of 10:90 (*v*/*v*) methanol/acetonitrile at a flow rate of 0.5 mL/min. The retention time for vitamin D_2_ was 2.1 min.

### 2.4. Participant Recruitment

Thirty-six healthy participants were recruited at Taipei Medical University, Shih Chien University, and the Family Medicine Health Check Center at Taipei Medical University Hospital. Inclusion criteria were as follows: aged 20 to 60 years, any gender, and able to consume one mushroom meal pack daily. Exclusion criteria included the following: (1) Individuals with any acute diseases such as infections, stroke, myocardial infarction, major surgery within the past three months, upper or lower gastrointestinal bleeding, and poorly controlled blood pressure or blood glucose. (2) Individuals with conditions such as malignant tumors, human immunodeficiency virus (HIV), cirrhosis or liver function exceeding three times the normal value (AST or ALT > 120 IU/L), abnormal kidney function (creatinine greater than 2.5 mg/dL), anemia (Hb < 9 g/dL), metabolic disorders other than diabetes (e.g., thyroid or parathyroid dysfunction), or previous abdominal surgery resulting in intestinal adhesions. (3) Pregnant or breastfeeding women. (4) Individuals taking steroids, vitamin D supplements, or hormones. (5) Participants who had had a previous allergy to mushrooms. (6) Participants with serum 25(OH)D levels > 30 ng/mL.

This study was approved by the Taipei Medical University Institutional Review Board (N202111054). This study has also been registered with ClinicalTrials.gov, with the registration number NCT05716698. The study was initiated after obtaining informed consent from participants who fully understood the study’s objectives, procedures, and the guidelines to be followed during the study.

### 2.5. Clinical Trial Design

Thirty-six healthy participants were randomly assigned to one of three groups in a parallel design: (1) a control group with no PC intervention (Control), (2) a group consuming 10 g of PUV-treated PC daily (PC-10 g), and (3) a group consuming 100 g of PUV-treated PC daily (PC-100 g). Each group consisted of 12 participants and underwent a four-week experimental intervention.

Prior to trial entry, participants who had been regularly taking any form of vitamin D supplements were excluded. Although a specific washout period was not implemented, baseline serum 25(OH)D levels were measured, and only individuals with levels below 30 ng/mL were included in the study, ensuring that all participants were either vitamin-D-insufficient or -deficient at the start of the trial.

Before and after the trial, participants were required to fast for at least eight hours and then undergo venous blood collection of 11 mL for biochemical testing and analysis of serum 25(OH)D_2_ and 25(OH)D_3_. During the intervention period, participants were reminded daily via the LINE messaging app to consume the PC meal packs and to report any adverse effects. Participants visited the laboratory weekly to pick up their PC meal packs and return any empty or uneaten packs, which helped to ensure compliance. We ensured that the compliance rate among the participants was nearly 100%, and none of the participants reported any adverse side effects. All participants maintained the same general activities and regular diet during the trial as before the trial.

### 2.6. Blood Biochemical Measurements

Blood biochemical analyses, including fasting plasma glucose, triglycerides, total cholesterol, low-density lipoprotein cholesterol (LDL-C), high-density lipoprotein cholesterol (HDL-C), aspartate aminotransferase (AST), alanine aminotransferase (ALT), blood urea nitrogen (BUN), creatinine, calcium, phosphorus, magnesium, high-sensitivity C-reactive protein (hs-CRP), glycated hemoglobin (HbA1c), and intact parathyroid hormone (iPTH), were performed using standardized methods at Yadong Medical Laboratory (Zhongli, Taiwan). Specifically, fasting plasma glucose was measured using a Beckman Coulter AU5800 automated chemistry analyzer (Beckman Coulter, Brea, CA, USA), and HbA1c was analyzed with a Premier Hb9210™ automated analyzer (Trinity Biotech, Wicklow, Ireland).

### 2.7. Serum 25(OH)D_2_ and 25(OH)D_3_ Analysis

During the study, 4 mL of whole blood was collected from the 36 participants at baseline and on day 28, and serum was separated for the measurement of 25(OH)D_2_ and 25(OH)D_3_, and the extraction preparation of blood samples followed the method by Chen et al. [19].

First, 0.5 mL of serum was mixed with 2 mL of ethanol and shaken for 15 s. Then, 2 mL of deionized water was added, and the mixture was shaken for another 15 s. Next, 3 mL of hexane was added and shaken for 1 min. The mixture was then centrifuged using a HITACHI Centrifuge CT6E (HITACHI, Japan) at 10,000 rpm for 10 min to collect the upper organic layer. The extraction process was repeated with an additional 3 mL of hexane, and the supernatant was collected. The two extracts were combined and freeze-dried using a Scanvac ScanSpeed 32 Teflon freeze-dryer. Prior to analysis, 100 μL of methanol was added to reconstitute the residue.

This study utilized the ultra-high-performance liquid chromatography–mass spectrometry (UHPLC-MS) method of analysis. The detection of 25(OH)D_2_ and 25(OH)D_3_ was performed using a Thermo Scientific Vanquish Horizon UHPLC system coupled with atmospheric pressure chemical ionization (APCI) and an ion trap analyzer, specifically, an Orbitrap Fusion Lumos tribrid mass spectrometer (Thermo Scientific). The UHPLC injection temperature was set at 5 °C, and the column used was an ACQUITY UPLC CSH C18, 1.7 µm (2.1 × 50 mm, Waters). The column temperature was set to 40 °C for separation. Mobile phase A was 25% acetonitrile (ACN), and mobile phase B was a mixture of ACN and methanol in a 3:1 ratio. The flow rate was set to 0.4 mL/min. Initially, the mobile phase ratio was 20% A and 80% B, which was maintained for 4 min. Then, the proportion of mobile phase B was increased to 100% and maintained for 1 min. At 5.1 min, the proportion of mobile phase B was decreased to 80%, and this ratio was maintained until the end of the run at 6.5 min. High-energy collision-induced dissociation was employed for the analysis. The ionization temperature was set at 350 °C, and the ion transfer temperature was set at 275 °C. The spray voltage was 4 µA in the positive mode, and the sheath gas flow rate was set to 45 arbitrary units. The monitoring time was 8 min.

### 2.8. Statistical Methods

All data are presented as mean ± standard deviation (SD). The Shapiro–Wilk test was used to assess the normality of the data distribution. Within-group comparisons before and after the intervention were conducted using paired *t*-tests. Differences between groups were analyzed using one-way analysis of variance (ANOVA) followed by Tukey’s post hoc Honestly Significant Difference (HSD) test. Statistical significance was set at *p* < 0.05. All analyses were performed using IBM SPSS Statistics, Version 19.

## 3. Results

### 3.1. Vitamin D_2_ Content in PUV-Light-Treated PC

The non-PUV-treated fresh PC contained extremely low levels of vitamin D_2_, all below 1 µg per 100 g of fresh weight, which is under the detection limit of UPLC. After PUV treatment, the PC had an average vitamin D_2_ content of 248 µg per 100 g. Following cooking, approximately 7.8% of the vitamin D_2_ was lost. Therefore, the mushroom meal packs provided to the participants contained an average of 248 µg of vitamin D_2_ per 100 g (Table 1).

### 3.2. Subjects Recruitment and Basic Characteristics of the Participants

**Figure 1:**
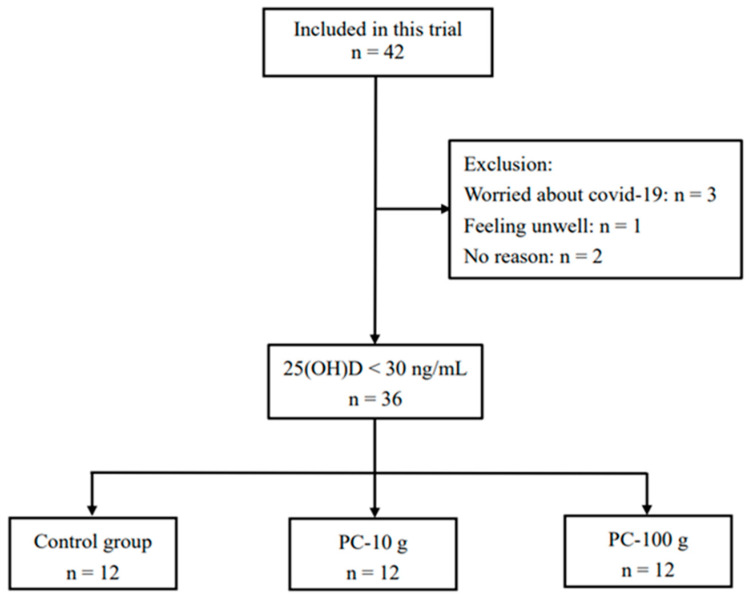
Flowchart of subject recruitment.

Recruitment for this study began in March 2022. By April 2022, a total of 42 participants were recruited. However, three participants withdrew before the experiment began due to concerns about the COVID-19 pandemic, one participant withdrew before starting the study due to health issues, and two participants withdrew without providing a reason. Ultimately, 36 participants (5 males and 31 females) met the inclusion criteria, signed the consent forms, and completed the study (Figure 1).

The basic characteristics of the participants are presented in Table 2. The results indicate that the control group (31.3 ± 12.9 yrs) had a significantly higher mean age compared to the PC-10 g group (21.9 ± 2.0 yrs), while the PC-100 g group (24.3 ± 5.7 yrs) showed no significant difference from the other two groups. There were no other statistical differences between the groups in terms of gender distribution, education level, or medical history.

### 3.3. Blood Biochemical Measurements of the Participants

This study randomly assigned 36 subjects into three groups: the control group (n = 12), the PC-10 g group (n = 12), and the PC-100 g group (n = 12). Blood biochemical parameters, including fasting plasma glucose (FPG), total cholesterol, triglycerides (TG), aspartate transaminase (AST), alanine transaminase (ALT), blood urea nitrogen (BUN), creatinine, high-sensitivity C-reactive protein (Hs-CRP), magnesium (Mg), calcium (Ca), and phosphorus (P), were measured at baseline (day 0) and after 28 days of intervention. The results, as shown in Table 3, demonstrated no significant changes in any parameters in the control group (*p* > 0.05). Similarly, in both the PC-10 g and PC-100 g groups, where the participants consumed 10 g and 100 g of PUV-treated PC daily, none of the measured biochemical parameters showed statistically significant differences between day 0 and day 28 (*p* > 0.05). Furthermore, in the PC-10 and PC-100 g group, which received a high dose of vitamin D_2_, no adverse effects were observed in the liver (AST, ALT) or kidney (BUN, creatinine) indicators, suggesting that the consumption of the UV-treated PC was safe and did not affect the blood biochemical parameters in the subjects.

### 3.4. Serum 25(OH)D_2_ and 25(OH)D_3_ Levels of Participants Before and After the Trial

**Figure 2:**
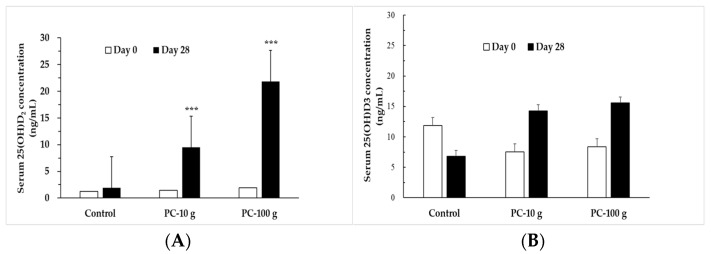
Serum 25(OH)D_2_ (**A**) and 25(OH)D_3_ (**B**) concentration in subjects before and after PC intervention, measured by LC-MS analysis. *** means *p* < 0.001.

The changes in serum 25(OH)D levels before and after the trial are shown in Figure 2. The results showed that before PC supplementation, the serum 25(OH)D_2_ levels of all subjects were very low. After the intervention, except for the no differences observed in the control group before and after intervention, the PC-10 g group showed a significant increase in serum 25(OH)D_2_ from 1.47 ± 1.42 ng/mL to 9.50 ± 7.10 ng/mL after four weeks (*p* < 0.001), while that of the PC-100 g group increased significantly from 1.94 ± 2.15 ng/mL to 21.82 ± 16.75 ng/mL (*p* < 0.001). The serum 25(OH)D_2_ levels significantly increased with the dosage of the supplement. However, there was no significant difference in serum 25(OH)D_3_ levels before and after supplementation. This indicates that consuming PUV-treated PC can significantly enhance the vitamin D nutritional status of healthy subjects.

### 3.5. Regulatory Effect of High-Dose PUV-Treated PC Supplementation on Serum Parathyroid Hormone Levels

**Figure 3:**
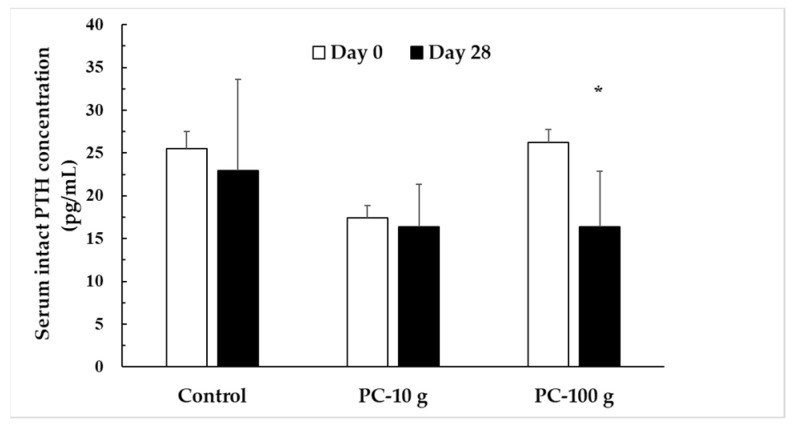
Serum intact PTH concentration in subjects before and after PC intervention. PTH, parathyroid hormone. * *p* > 0.001.

A decrease in 25(OH)D reduces calcium absorption, leading to lower serum calcium levels, which triggers the release of parathyroid hormone (PTH). Notably, high-dose PC supplementation significantly increased serum 25(OH)D_2_ levels and decreased serum intact PTH levels by 37.6% (*p* = 0.013). This finding suggests that high-dose PC may improve PTH levels, likely due to the enhanced intake of vitamin D_2_ (Figure 3).

## 4. Discussion

Vitamin D plays a vital role in the body, including enhancing the intestinal absorption of calcium, magnesium, and phosphorus. Vitamin D deficiency has become a global concern and is associated with various diseases, including bone disorders. However, large-scale vitamin D supplementation trials have yielded mixed results, highlighting the need for further research to clarify its specific effects [20]. A meta-analysis by Cui et al., incorporating data from 7.9 million participants across 81 countries, revealed that between 2000 and 2022, the global prevalence of vitamin D levels below 75 nmol/L was approximately 76.6%. The study found that Africa had the lowest prevalence, while the eastern Mediterranean region exhibited the highest. Higher prevalence rates were observed among females, during winter and spring seasons, in high-latitude regions, and in low- to middle-income countries [21]. According to the Korea National Health and Nutrition Examination Survey (KNHANES) data from 2016 to 2019, the average dietary intake of vitamin D among Koreans is significantly lower than the recommended daily intake, averaging only 3.13 micrograms per day. Further analysis of dietary sources reveals that fish and shellfish are the primary contributors, accounting for 61.59%, followed by eggs (17.75%), meat (8.03%), milk (4.25%), legumes (3.93%), and grains (3.84%). This survey also highlights that animal-based sources remain the predominant means of obtaining vitamin D for Koreans [22]. Lee et al. also found similar results in Taiwan [23]. Jiang et al. found that 83% of Chinese adults aged 18 to 65 had serum 25(OH)D_3_ levels below 30 ng/mL, with 41.9% showing deficiency (10–19 ng/mL) and 8.4% severe deficiency (<10 ng/mL). Women, winter and spring seasons, northern regions, and younger age groups were associated with lower vitamin D levels [24]. The most recent National Nutrition and Health Survey in Taiwan (NAHSIT, 2017–2020) indicated that adult males consumed only 52% to 57% of the adequate intake (AI) for vitamin D recommended in the eighth edition of the Dietary Reference Intakes (DRIs), while females consumed just 38% to 49%. This inadequate intake was reflected in the serum 25(OH)D_2_ levels of the participants in our study, which were consistently found to be extremely low before the intervention.

Fungi-derived sources of vitamin D offer advantages such as environmental friendliness and lower costs, which may contribute to improving human vitamin D nutritional status. Wild mushrooms can naturally absorb UV light, typically containing vitamin D_2_. However, modern farming practices, which focus on producing high-quality mushrooms, often involve growing mushrooms under controlled light, humidity, and temperature conditions. As a result, vitamin D_2_ levels in these cultivated mushrooms are usually undetectable [11]. Traditional UV irradiation methods are effective in producing high concentrations of vitamin D_2_ and have also been shown to enhance antioxidant capacity [25]; however, they often lead to issues such as blackening, exudation, and reduced shelf-life of fresh mushrooms due to prolonged UVB exposure. In contrast, the pulsed UV light (PUV) method described by Kalaras et al. does not affect the color or quality of fresh mushrooms, making it an optimal approach for producing mushrooms with high concentrations of vitamin D_2_ [10,26].

Several studies have demonstrated that vitamin D_2_ in mushrooms is bioavailable. Urbain et al. conducted a single-blind, randomized, placebo-controlled trial over five weeks with 26 healthy adults whose 25(OH)D levels were below 20 ng/mL. The participants were randomly assigned to one of three groups: one receiving UV-irradiated mushroom soup (providing 28,000 IU per week), one receiving the same dose of vitamin D_2_ supplements, and a placebo group (non-UV-irradiated mushroom soup). At the end of the trial, the serum 25(OH)D levels significantly increased in the UV-irradiated mushroom soup group, showing similar effectiveness to the vitamin D_2_ supplements in raising 25(OH)D levels [27]. Stephensen et al. found that after providing participants with UV-untreated mushroom meals (control), UV-irradiated mushroom meals containing 352 IU or 684 IU of vitamin D_2_, and purified ergocalciferol (1128 IU) plus untreated mushrooms for six weeks, all groups except the control group showed increased levels of 25(OH)D_2_. However, there was a decrease in serum 25(OH)D_3_ levels across all groups consuming vitamin D_2_. The reduction in 25(OH)D_3_ may have offset the increase in 25(OH)D_2_ levels [28]. However, more research has confirmed that whether in capsule form, added to foods, or obtained from UV-irradiated mushrooms, vitamin D_2_ can effectively increase serum levels of 25(OH)D [8,29,30]. Our study shows that after 4 weeks of consuming PC treated with PUV light, the participants had a significant increase in serum 25(OH)D_2_ levels, while 25(OH)D_3_ levels not only did not decrease but also showed a slight increasing trend. Since we only asked the participants to avoid mushroom-related foods during the experiment and did not place restrictions on foods rich in vitamin D3, such as fish, eggs, and milk, and since we encouraged them to maintain their usual daily activities and food intake, this may have contributed to the maintenance of serum 25(OH)D_3_ levels among the participants. Kleftaki et al.’s study demonstrated that a *Pleurotus eryngii* mushroom snack enhanced with vitamin D2 improved glucose regulation, reduced body weight, fat, waist, and hip circumferences, and increased 25(OH)D_2_ levels in metabolically unhealthy patients. It also lowered LDL, SGOT, IL-6, and ox-LDL levels while enhancing overall physical health, showcasing antidiabetic, antiobesity, anti-inflammatory, and antioxidant benefits [31].

In this study, we also observed an interesting result: the PC-100 g group exhibited a significant decrease in serum intact PTH (I-PTH) levels. Some studies have observed a negative relationship between 25(OH)D levels and PTH levels [19,32]. According to physiological paradigms, vitamin D promotes calcium absorption to maintain serum calcium levels. When vitamin D is deficient, serum calcium levels may decrease, which stimulates increased PTH secretion to maintain calcium homeostasis, often at the cost of increased bone turnover. However, in this study, all the participants maintained serum calcium levels within the normal range before and after the trial, with no significant changes observed across the groups. Despite the reduction in PTH levels in the PC-100 g group after consuming a high dose of PUV-treated PC, serum calcium levels remained stable. While this aligns with the theoretical relationship between vitamin D and calcium homeostasis, further studies are required to confirm whether PUV-treated PC directly aids calcium absorption or affecpts markers of bone turnover.

Revious animal studies have shown that consuming vitamin-D-enriched edible mushrooms can enhance serum vitamin D levels, lower PTH concentrations, and improve the osteoid area while slowing trabecular bone loss in the femur [33,34]. However, without direct evidence from our study on changes in calcium levels or serum markers of bone formation and resorption, any claims about the impact of PUV-treated PC on calcium homeostasis remain speculative and should be interpreted with caution.

Fulgoni et al. analyzed data from the National Health and Nutrition Examination Survey (NHANES) 2011–2016 and found that incorporating a serving of UV-exposed mushrooms (providing 5 µg of vitamin D per serving) to the daily diet nearly doubles vitamin D intake (an increase of 98–104%). This dietary change significantly reduces the risk of vitamin D deficiency and simultaneously increases the intake of various other nutrients [35]. A dietary model also indicates that consuming four servings per week of UV-exposed mushrooms can help most Australian adults meet their vitamin D recommendations [36]. Therefore, UV-exposed mushrooms represent a crucial tool in the global strategy for addressing vitamin D deficiency.

Initially, our study was designed with 10 g of PC (approximately 20 µg of vitamin D) as the low dose and a maximum of one serving per day (100 g) of mushrooms (approximately 200 µg of vitamin D) as the high dose. The results demonstrated that the high dose more effectively improved vitamin D status, and no adverse side effects were reported among the participants. However, this was a short-term trial, and long-term safety assessments are still needed. Recently, the European Food Safety Authority’s Panel on Nutrition, Novel Foods, and Food Allergens (NDA) reviewed the production process, composition, and specifications of *Agaricus bisporus* mushroom powder that had been exposed to ultraviolet (UV) irradiation to induce vitamin D_2_ at levels ranging from 245–460 µg/g as a novel food (NF) according to EU regulations. The panel considers that the total intake of vitamin D from novel foods (NFs), background diets, and fortified foods is safe when it is below the upper limits (ULs) for vitamin D previously established by the NDA panel for children, adolescents, and adults (50 µg/day and 100 µg/day, respectively) [37]. This recommendation may also be referenced in the future when promoting PUV-treated PC.

This study has several limitations. The relatively small sample size and short 4-week intervention period restricted the observation of long-term physiological effects. Additionally, challenges from the COVID-19 pandemic, including participant recruitment and follow-up restrictions, further constrained the study’s scope and sample size. Another limitation is the approximately 8% loss of vitamin D in the PUV-treated PC mushrooms after steaming, with the preservation of vitamin D using other cooking methods remaining unclear. Furthermore, although reductions in iPTH levels were observed, serum calcium levels in all groups remained within the normal range, indicating stable calcium homeostasis. As a result, this study could not provide conclusive evidence on the effects of PUV-treated PC on calcium metabolism or bone health markers. These limitations highlight the importance of conducting larger-scale and longer-term studies in the future to validate the findings and evaluate the long-term benefits of PUV-treated PC.

Cultivation trials demonstrate that PC mushrooms can thrive under a wide range of conditions, including growth on broad-leaf hardwood sawdust at a pH of 6 and temperatures between 20–32 °C. This adaptability makes them suitable for year-round cultivation across all regions of Taiwan. In the future, PC mushrooms could serve as a natural, sustainable, and fungi-derived food to address vitamin D deficiencies in Taiwan and Asia.

## 5. Conclusions

Based on the research findings, consuming *Pleurotus citrinopileatus* (PC) treated with PUV effectively increased serum 25(OH)D_2_ levels in a dose-dependent manner, improving the participants’ vitamin D nutritional status without any observed adverse side effects. These results highlight the potential of PUV-treated PC as a valuable source of vitamin D, contributing to advancements in processing, storage preservation, and addressing vitamin D deficiency-related disease control.

## Figures and Tables

**Table 1:** Vitamin D_2_ content in Pleurotus citrinopileatus exposed to PUV across different batches.

Batches	Fresh PC Mushroom (µg /100 g)	Cooked PC Mushroom (µg /100 g)	Cooking Loss Rate (%)
First Batch	218.1 ± 0.33	199.7 ± 0.75	8.5
Second Batch	278.3 ± 11.6	252.1 ± 14.1	9.4
Third Batch	250.9 ± 17.8	235.2 ± 5.9	6.3
Fourth Batch	245.4 ± 10.1	228.6 ± 1.5	6.9
Average	248.2 ± 10.0	228.9 ± 5.6	7.8

Data in the table are expressed as mean ± SD.

**Table 2:** Blood biochemical measurements of the healthy subjects.

Characteristic	Control (*N* = 12)	PC-10 g (*N* = 12)	PC-100 g (*N* = 12)	*p*
Age (years)	31.3 ± 12.9	21.9 ± 2.0	24.3 ± 5.7	0.024 *
Male/female (n)	2/10	1/11	2/10	0.807
Education				
High school (n)	1	0	0	
College (n)	11	12	12	
Medical history				
Hypertension (n)	1	0	0	
Hyperlipidemia (n)	1	1	2	
Diabetes (n)	0	0	0	

Data are expressed as mean ± standard deviation or numbers and analyzed by one-way ANOVA followed by Tukey’s post hoc Honestly Significant Difference (HSD) test. *p* < 0.05 was considered as statistically significant and is shown with an asterisk.

**Table 3:** Blood biochemical measurements of the subjects.

Items	Control (*N* = 12)			PC-10 g (*N* = 12)			PC-100 g (*N* = 12)		
	Day 0	Day 28	*p*	Day 0	Day 28	*p*	Day 0	Day 28	*p*
FPG (mg/dL)	81.8 ± 5.8	81.7 ± 4.1	0.936	84.3 ± 5.3	82.4 ± 6.2	0.424	81.7 ± 4.7	79.7 ± 7.4	0.436
Cholesterol (mg/dL)	187.8 ± 40.7	179.2 ± 28.9	0.553	172.1 ± 32.7	180.3 ± 36.7	0.567	200.1 ± 35.3	198.6 ± 38.8	0.922
TG (mg/dL)	85.6 ± 54.5	89.3 ± 57.0	0.874	98.3 ± 110.9	87.2 ± 81.4	0.783	83.3 ± 46.2	77.3 ± 21.8	0.688
AST (U/L)	17.8 ± 5.2	20.2 ± 5.5	0.294	24.3 ± 22.9	19.3 ± 5.9	0.464	17.8 ± 5.1	20.2 ± 6.1	0.321
ALT (U/L)	13.7 ± 5.1	14.4 ± 5.7	0.736	15.9 ± 12.06	16.4 ± 15.61	0.931	17.2 ± 17.1	19.5 ± 19.7	0.759
BUN (mg/dL)	14.4 ± 3.2	13.7 ± 2.9	0.554	11.3 ± 2.0	11.1 ± 3.1	0.877	11.7 ± 2.1	12.5 ± 2.4	0.370
Creatinine (mg/dL)	0.7 ± 0.1	0.7 ± 0.1	0.611	0.7 ± 0.1	0.6 ± 0.1	0.352	0.7 ± 0.1	0.7 ± 0.1	0.442
Hs-CRP (mg/L)	0.57 ± 0.57	1.27 ± 2.17	0.290	0.36 ± 0.30	0.48 ± 0.38	0.387	0.74 ± 0.96	0.52 ± 0.53	0.481
Mg (mg/dL)	2.07 ± 0.14	1.97 ± 0.11	0.059	1.99 ± 0.14	1.98 ± 0.17	0.898	2.03 ± 0.16	1.91 ± 0.17	0.095
Ca (mg/dL)	9.42 ± 0.32	9.18 ± 0.34	0.093	9.40 ± 0.31	9.35 ± 0.23	0.655	9.42 ± 0.26	9.40 ±0.26	0.878
P (mg/dL)	3.98 ± 0.41	3.70 ± 0.55	0.166	4.15 ± 0.49	3.87 ± 0.38	0.126	3.94 ± 0.38	3.88 ± 0.29	0.679

Data are expressed as mean ± standard deviation and were analyzed by paired *t*-test; FPG, fasting plasma glucose; TG, triglycerides; AST, aspartate transaminase; ALT, alanine transaminase; BUN, blood urea nitrogen; Hs-CRP, high-sensitivity C-reactive protein. *p* < 0.05 was considered as statistically significant.

## Data Availability

The original contributions presented in this study are included in the article/Supplementary Materials. Further inquiries can be directed to the corresponding author.

## Supplementary Materials

The following supporting information can be downloaded at: https://www.mdpi.com/article/10.3390/foods14020259/s1, Supplementary File S1: CONSORT 2010 checklist of information to include when reporting a randomised trial.
